# Highly gate-tuneable Rashba spin-orbit interaction in a gate-all-around InAs nanowire metal-oxide-semiconductor field-effect transistor

**DOI:** 10.1038/s41598-017-01080-0

**Published:** 2017-04-19

**Authors:** K. Takase, Y. Ashikawa, G. Zhang, K. Tateno, S. Sasaki

**Affiliations:** 1 NTT Basic Research Laboratories, NTT Corporation, 3-1 Morinosato-Wakamiya, Atsugi, Kanagawa 243-0198 Japan; 2 Department of Physics, Tohoku University, 6-3 Aramaki Aza Aoba, Aoba-ku, Sendai 980-8578 Japan

## Abstract

III-V semiconductors have been intensively studied with the goal of realizing metal-oxide-semiconductor field-effect transistors (MOSFETs) with high mobility, a high on-off ratio, and low power consumption as next-generation transistors designed to replace current Si technology. Of these semiconductors, a narrow band-gap semiconductor InAs has strong Rashba spin-orbit interaction, thus making it advantageous in terms of both high field-effect transistor (FET) performance and efficient spin control. Here we report a high-performance InAs nanowire MOSFET with a gate-all-around (GAA) structure, where we simultaneously control the spin precession using the Rashba interaction. Our FET has a high on-off ratio (10^4^~10^6^) and a high field-effect mobility (1200 cm^2^/Vs) and both values are comparable to those of previously reported nanowire FETs. Simultaneously, GAA geometry combined with high- κ dielectric enables the creation of a large and uniform coaxial electric field (>10^7^ V/m), thereby achieving highly controllable Rashba coupling (1 × 10^−11^ eVm within a gate-voltage swing of 1 V), i.e. an operation voltage one order of magnitude smaller than those of back-gated nanowire MOSFETs. Our demonstration of high FET performance and spin controllability offers a new way of realizing low-power consumption nanoscale spin MOSFETs.

## Introduction

The high electron mobility of III-V semiconductors makes them good candidates for the development of field-effect transistors that can be operated with high speed, a high on-off ratio, and a low power consumption. Of these semiconductors, those showing band structures with large spin-orbit splitting have been independently attracting great interest in relation to spin FET applications^1^. The large band splitting is mostly associated with the Rashba spin-orbit interaction (SOI) generated with an electric field induced by structural inversion asymmetry. The Rashba SOI is given by the Hamiltonian, *H* = *e*α_0_ ∙ (σ × *k*), where *e* is an elementary charge, α_0_ is a Rashba coefficient determined from the band structure of a bulk material, σ is the Pauli matrix, *k* is the electron wave vector and *E* is the electric field vector^2–4^. The Rashba coupling parameter given by α ≡ α_0_ *eE* is an important index as a measure of modulating electron spin, and increasing and controlling α with the gate voltage has been a focus of attention.

To obtain better electric-field control of the Rashba SOI, III-V semiconductors such as GaAs/AlGaAs, GaInAs/InP, InAs, InSb and InGaAs have been investigated for various structures including two-dimensional electron gas (2DEG) in heterostructures^5–7^, quantum wells (QW)^8–10^ and quantum wires^11^ using a top-down microfabrication process. These studies reported that Rashba parameters range from α = 0.3 × 10^−11^ to 1 × 10^−11^ eVm (refs 6–11) and have a gate voltage *V*_g_ tunability of ~1.4 × 10^−12^ eVm/V (refs 5–9). On the other hand, InAs nanowires with surface electron confinement potential in a sub-micron width have been examined mostly in the form of conventional bottom- or top-gated devices^12–16^. They have shown a larger α (1 × 10^−11^–3 × 10^−11^ eVm) but the *V*_g_ tunability was as small as that of former reports using a top-down approach^7–11^ within a gate voltage range of 0–20 V. Recently, Liang *et al*.^17^ reported ion-gated InAs nanowire device, exhibiting *V*_g_ tunable efficiency more than ten times higher than previously reported^13, 14, 16, 18^. This marked progress in efficiency brought about low-gate voltage operation leading to low-power consumption. However, since ion-gated device requires very long response time, a prototype device employing standard MOS design that excels in operation speed is critically needed.

Here, we report high gate-tunability of the Rashba SOI in an InAs nanowire MOSFET employing gate-all-around (GAA) geometry^19^, in which gate-induced electric field is more enhanced and more uniform than those in conventional bottom- or top-gated nanowire devices^13–16^, multigated nanowires^18^, and Ω-shape (partially coaxial) gated devices^20–22^. The Rashba parameter that we obtained by weak antilocalization measurements is 0.6 × 10^−11^–2 × 10^−11^ eVm, and the gate voltage tunability is 1.2 × 10^−11^–2.4 × 10^−11^ eVm/V, the latter being ten times larger than that obtained for various types of III-V semiconductors including InAs nanowire MOSFETs^6–16^. This is also comparable to the best *V*_g_ tunability achieved for an ion-gated InAs nanowire FET^17^. In addition to the excellent *V*_g_ tunability of the Rashba SOI, our device exhibits excellent FET characteristics including a high on-off ratio (10^4^~10^6^) and a high field-effect mobility (1200 cm^2^/Vs). As MOSFETs have faster responses than ion-gated devices, which normally require considerable time for electric double layer stabilization^23^, our demonstration of both the excellent FET performance and high tunability of the Rashba SOI in a small *V*_g_ range could lead to the development of a practical spin nanowire MOSFET with low power consumption that is compatible with the currently used Si transistor platform.

## Results

**Figure 1:**
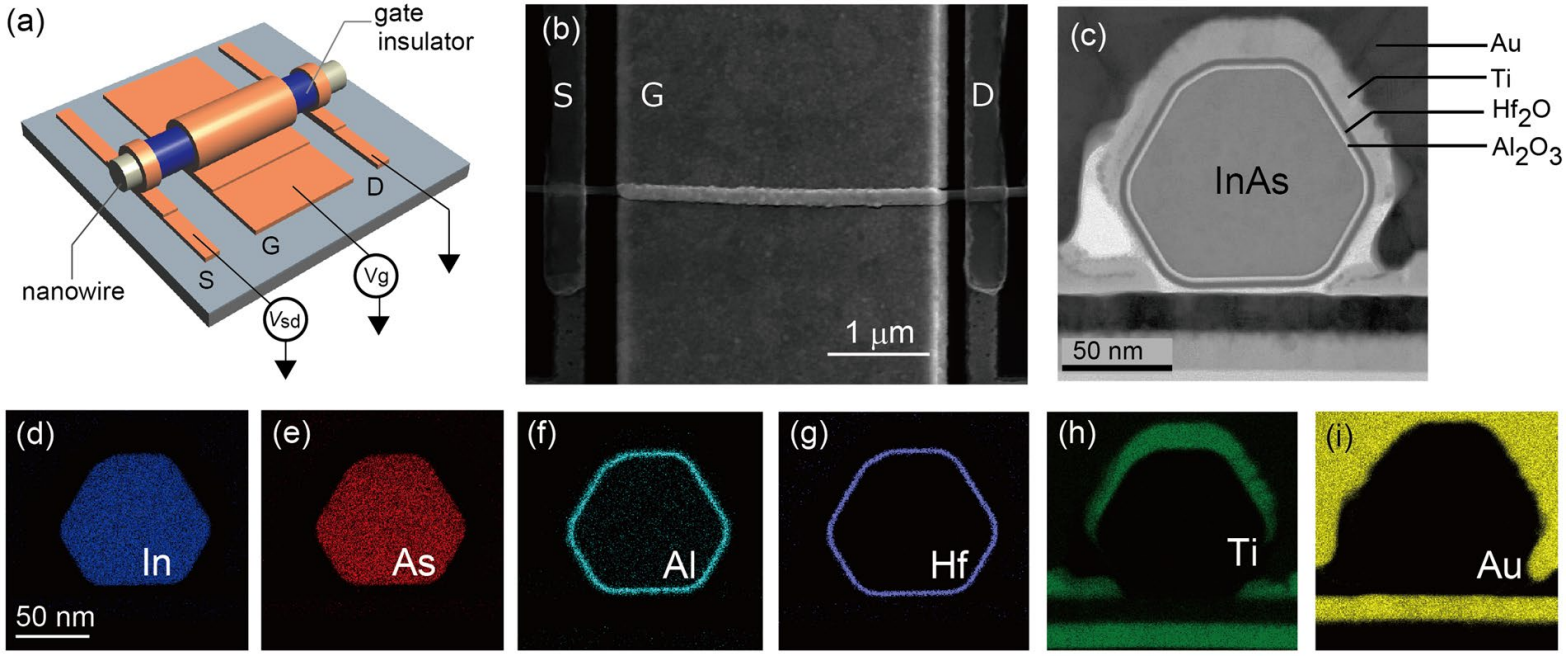
(**a**) Schematic illustration of our InAs nanowire GAA MOSFET together with circuit set up. (**b**) Top view of device used in this paper and (**c**) cross-sectional view of our typical device. (**d**–**i**) EDS images of device shown in (**c**). (**d**–**i**) correspond to the elemental mapping for (**d**) indium, (**e**) arsenic, (**f**) aluminium, (**g**) hafnium, (**h**) titanium, and (**i**) gold.

Figure 1(a) is a schematic illustration of our GAA InAs nanowire FET, which we fabricated using a similar method to the one we used for our previous nanowire FETs^24, 25^. GAA geometry, which is also called surrounding gate^26^ or wrap-gate^27^ geometry, has been used not only to induce a uniform electric field but also to suppress the short-channel effect of transistors^28^ with an improvement in nanowire FET performance^29, 30^. To obtain a high carrier density and thus induce a strong electric field, we used the high-κ gate dielectrics of Al_2_O_3_/HfO_2_ (2 nm/4 nm) grown by atomic layer deposition (ALD). The InAs nanowire coated with the above dielectrics was deposited on a pre-patterned substrate and then gate metal was evaporated onto the nanowire. This two-stage deposition of gate metal allows us to fabricate a GAA structure. As shown in Fig. 1(b), our sample is covered by the gate electrode over 90% of the channel length, which allows us to ignore the contributions of the ungated regions (for details see Method). Figure 1(c) shows a TEM image of a cross-section of a typical nanowire FET. We find that layered gate dielectrics and GAA geometry are formed according to our MOSFET design. These structures are also examined with energy dispersive X-ray spectrometry (EDS). The false colour images in Fig. 1(d–i) rule out any significant migration or diffusion of the deposited elements or contamination during the device processing along the entire channel.

**Figure 2:**
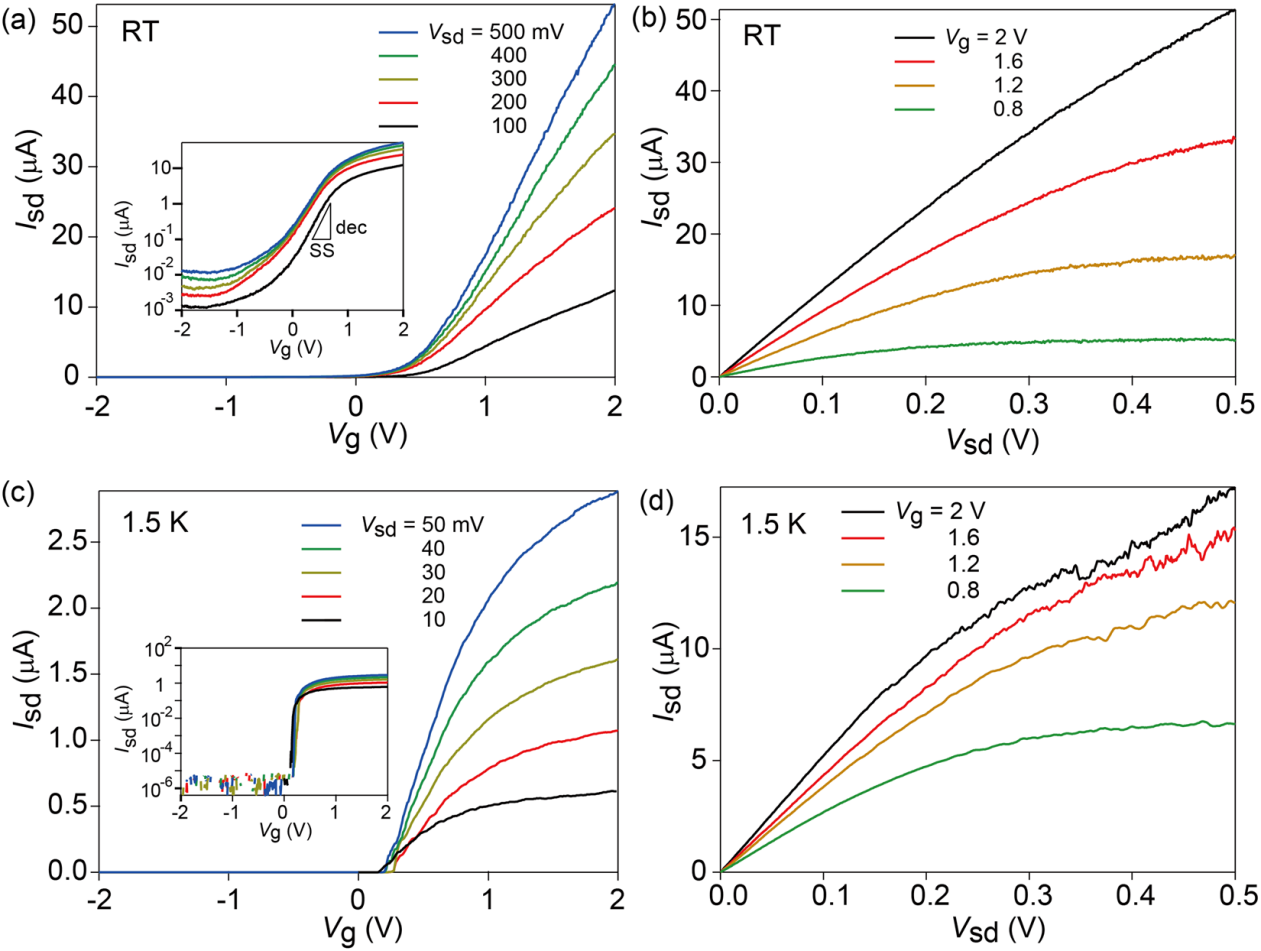
(**a**,**c**) Transfer characteristics obtained for various *V*_sd_ values at (**a**) room temperature and (**c**) 1.5 K. The insets show the log-scale of the plots, and the subthreshold slope. (**b**,**d**) Output characteristics obtained for various *V*_g_ values at (**b**) room temperature and (**d**) 1.5 K.

We first describe FET operation at various temperatures. Figure 2(a) shows the transfer characteristics of the device measured at room temperature for different source drain voltages *V*_sd_ of 100 to 500 mV. As shown in the inset, the subthreshold slope (SS) and on-off ratio are 350 mV/dec and over 10^4^ at room temperature (RT). Here SS is defined as *dV*_g_/*d*log*I*_sd_ with the source-drain current *I*_sd_. While the SS values for our typical devices fabricated in the same manner usually exceed 200 mV/dec at RT, which is larger than the ideal RT limit of 60 mV/dec, the on-off ratio exhibits good performance and is generally higher than ~10^4^. When we decrease the measurement temperature to 1.5 K, the SS and on-off ratio are greatly improved to 25 mV/dec and 10^6^, respectively, as shown in Fig. 2(c). The high on-off ratio at RT and 1.5 K are comparable to the excellent previously reported values for GAA InAs nanowires^24, 25, 31–33^ and GAA InGaAs nanowires^34^. Moreover, steep increase in *I*_sd_ within *V*_g_~1 V indicates that our GAA device is operated at lower voltage than conventional back-gated nanowire FETs with cylinder-on-plane (COP) geometry^13–16^. Figure 2(b,d) show the output characteristics for various *V*_g_ values measured at RT and 1.5 K, showing that a good saturation is obtained within a *V*_sd_ of 0.5 V.

**Figure 3:**
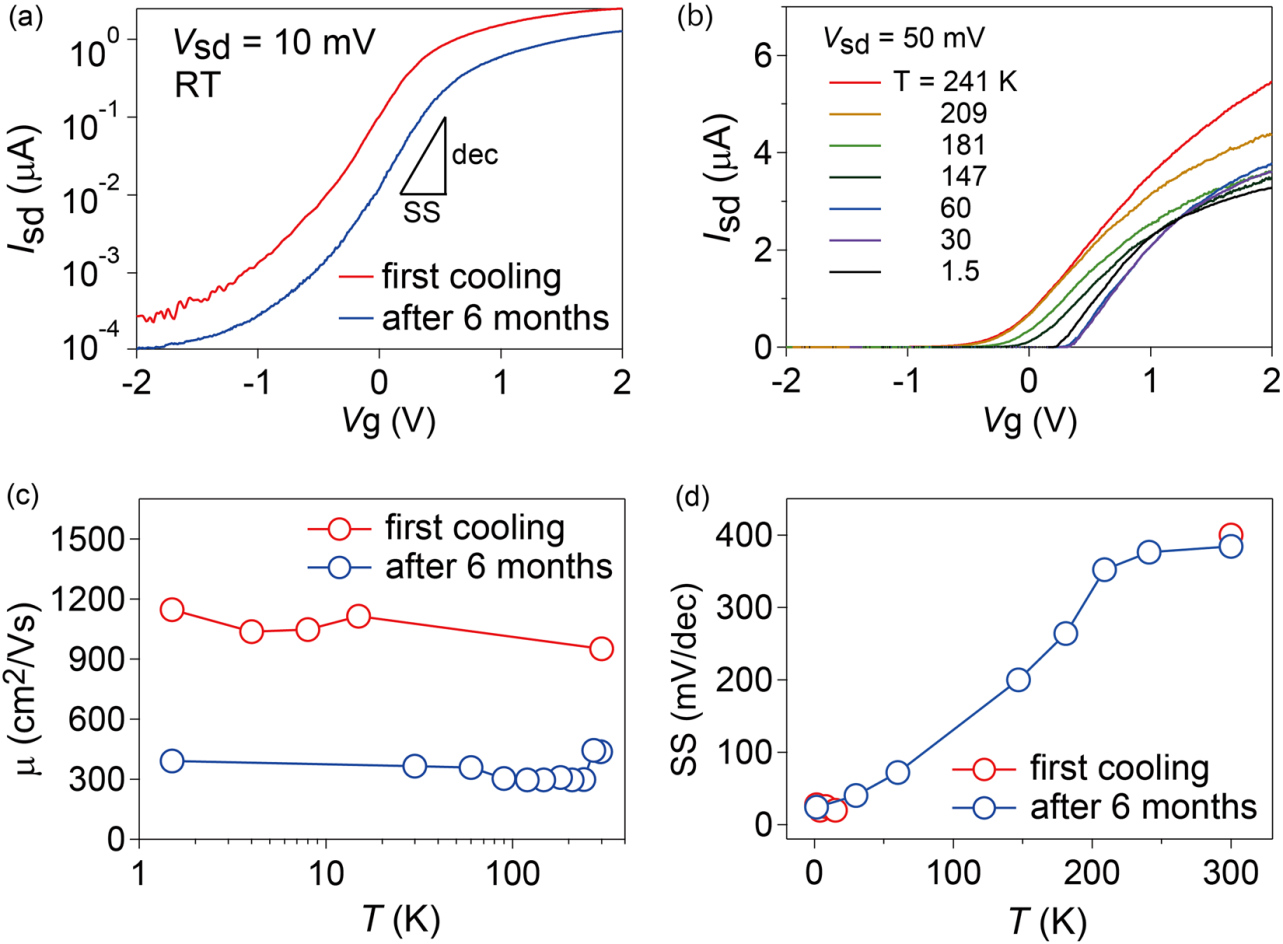
(**a**) Transfer characteristics of the device measured for the first cooling and after several coolings performed over 6 months. The device was stored in ambient conditions for 6 months when not in use. (**b**) Transfer characteristics of the device measured for various temperatures at *V*_sd_ = 50 mV. (**c**) Field-effect mobility and (**d**) SS values of the device plotted as a function of temperature for the first cooling and for the cooling after 6 months interval.

To investigate how robust our FET is under ambient conditions, we compare the same device in different measurement runs. Figure 3(a) compares the device transfer characteristics measured before the first cooling with those measured after 6 months, during which time the sample was stored in ambient air when not in use. Although reduction in *I*_sd_ is accompanied by a reduction in the on-off ratio from 2 × 10^4^ to 1 × 10^4^, we observe no notable change in SS values between the two cases. Moreover, our GAA device shows robust and clear transfer characteristics for various temperatures down to 1.5 K [Fig. 3(b)] after 6 months interval. We note that the data shown in Fig. 2(a–d) were measured after several cooling cycles, indicating that our FET performs well even after being affected by thermal cycles and the ambient conditions.

We next compare the field-effect mobility μ for the two cases and examine the temperature dependence. μ is given by $\frac{{{L}_{g}}^{2}}{C}\frac{d}{d{V}_{g}}(\frac{{I}_{sd}}{{V}_{sd}})$, where *L*_g_ is a gate length of 3.3 μm, *C* is a gate capacitance of 2.29 × 10^−14^ F, and *V*_sd_ is the source-drain bias. The sample exposed to the first thermal cycle shows mobilities of 1000 cm^2^/Vs at RT and 1200 cm^2^/Vs at 1.5 K, as shown in Fig. 3(c). Our device shows less *T* dependence than other InAs nanowire GAA devices^32, 33^. Indeed, many of our GAA devices possess mobilities of 1000–1500 cm^2^/Vs at room temperature. The value is one order of magnitude higher than that of a previously reported InAs GAA device using the gate dielectrics of HfO_2_ (~109 cm^2^/Vs)^31^, and comparable to single-crystalline and pure-phase InAs nanowire with GAA geometry^33^ (1500 cm^2^/Vs) and high-mobility InGaAs nanowire FETs (1030 cm^2^/Vs)^34^. However, after several thermal cycles and long-time storage under ambient conditions, the mobility decreased to around 400 cm^2^/Vs, which is nevertheless higher than the mobility of a high-κ gated MoS_2_ 2D transistor^35^ or a Si nanowire FET^36^. The decreased mobility may be attributed to increase in access resistance resulting from the nanowire segment that is not coated by the gate metal, possibly due to impurities adhered to that segment by repeated thermal cycles or during sample storage. Therefore, the decrease is merely in the extrinsic mobility, not the intrinsic one. This is also supported by the fact that SS after 6 months, which shows linear temperature dependence that is characteristic to standard FETs [Fig. 3(d)], has no notable difference from SS for the first cooling from room temperature to 1.5 K, indicating that surface states of the nanowire under the gate electrode are expected to be unaffected. In this paper, we use data obtained for the sample when it had a field effect mobility of ~400 cm^2^/Vs unless otherwise stated. However, we emphasize that gate efficiency on the nanowire channel was not degraded during 6 months, as is seen from virtually unchanged SS values. This is also consistent with the results obtained by magnetotransport measurements as we discuss later, in which we confirm that the gate controllability of the Rashba parameter was not degraded after 6 months.

**Figure 4:**
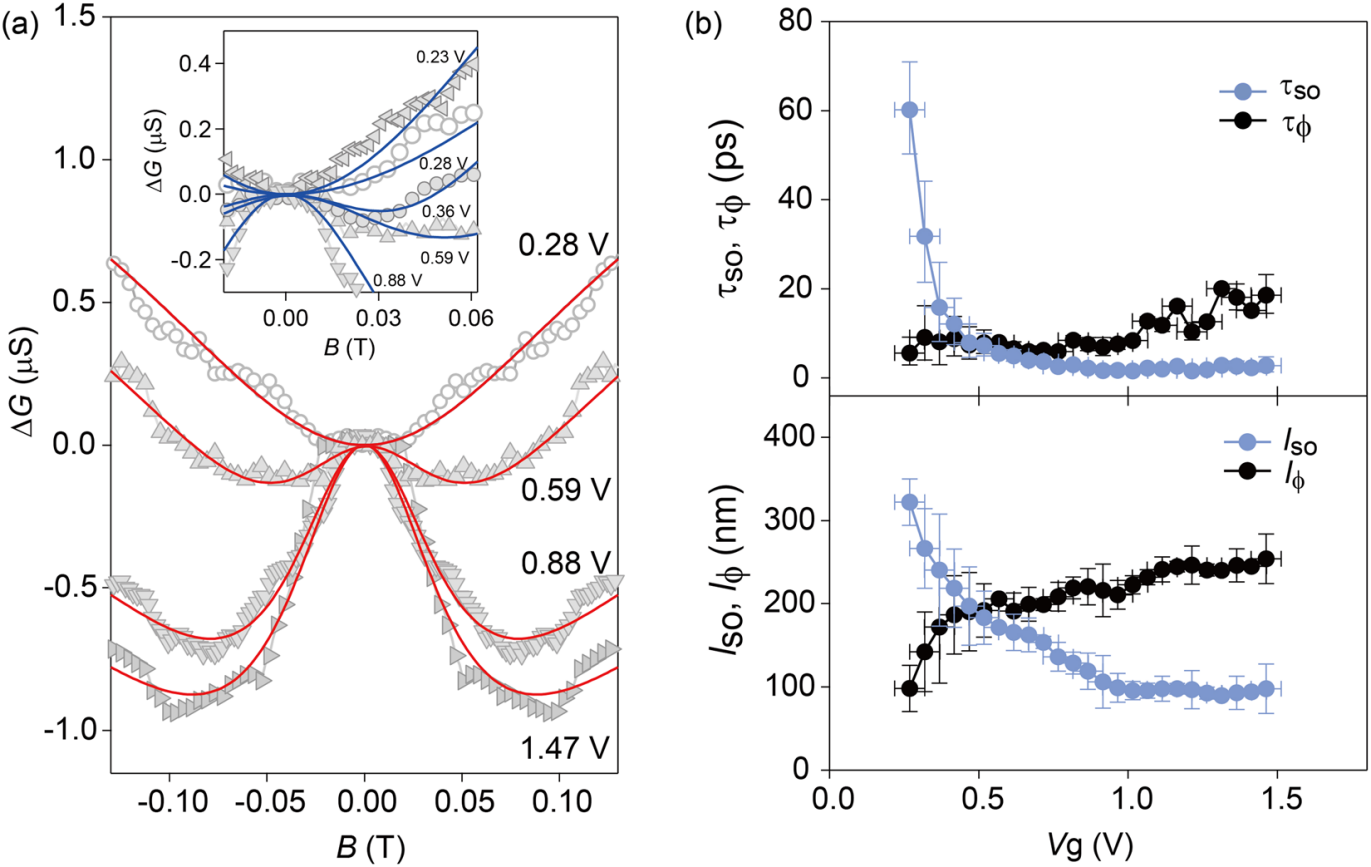
(**a**) Correction of conductance Δ*G* ≡ Δ*G*(B) − Δ*G*(0) as a function of magnetic field *B* for various gate voltages. The solid lines are fits using one-dimensional weak antilocalization model. The inset shows more data set near weak localization/weak antilocalization crossover. (**b**) *V*_g_ dependence of spin-orbit relaxation length *l*_so_ and phase coherence length *l*_ϕ_ extracted from Δ*G* shown in (**a**) (bottom) and corresponding relaxation time τ_so_ and τ_ϕ_ vs. *V*_g_ (top).

Having examined the FET performance of our device, we then investigated the effects of a spin-orbit interaction by conducting magnetotransport measurements at 1.5 K. Figure 4(a) shows the correction of magnetoconductance (ΔG ≡ Δ*G*(B) − Δ*G*(0)) as a function of a magnetic field (*B*), where the magnetoconductance was deduced from the two-terminal dc-transport at *V*_sd_ = 10 mV. The data have been smoothed over *V*_g_ ± 15 mV and *B* ± 15 mT to exclude universal conductance fluctuations or other random fluctuations caused by impurities, as in refs 14, 16. In addition, our data are further averaged with respect to the reversed magnetic field sweep direction to fit the data with better accuracy as described below. As *V*_g_ increases, *B* dependence of Δ*G* changes from a dip to a peak, indicating a crossover from weak localization to weak antilocalization^37, 38^, which occurs for conducting channels in a variety of materials and devices^9, 39, 40^ in the presence of a strong spin-orbit interaction.

Such a crossover from weak localization to weak antilocalization has also been observed for various types of InAs FETs^12–17^, where spin-orbit interaction is considered to be the Rashba SOI originating from a strong electric field. These devices have a mean free path shorter than the nanowire diameter, indicating that an electrical channel in a nanowire can be reasonably analysed in the framework of the disordered one-dimensional weak antilocalization model reported in ref. 38,

1 $${\rm{\Delta }}G=-\frac{2{e}^{2}}{h{L}_{g}}[\frac{3}{2}{(\frac{1}{{l}_{\varphi }^{2}}+\frac{4}{3{l}_{so}^{2}}+\frac{1}{D{\tau }_{B}})}^{-1/2}-\frac{1}{2}{(\frac{1}{{l}_{\varphi }^{2}}+\frac{1}{D{\tau }_{B}})}^{-1/2}]$$

where *h* is Planck constant, *L*_g_ is the gate length, *l*_ϕ_ is the phase coherence length, *l*_so_ is the spin-orbit relaxation length, *D* is the diffusion constant, and τ_B_ is the magnetic relaxation time. Here τ_B_ is given by

2 $${\tau }_{B}=\frac{3{{l}_{B}}^{4}}{{W}^{2}D}$$

with *l*_B_ being the magnetic length given by ${l}_{B}=\sqrt{h/(2\pi eB)}$. Note that using this relation reduces fitting parameters to only *l*_so_ and *l*_ϕ_.

Our device has a typical mean free path of 12 nm, which is smaller than the nanowire diameter of 100 nm. Therefore, the use of Eq. (1) is justified, as plotted by the solid lines in Fig. 4(a), which fit well with our data. *l*_so_ and *l*_ϕ_ are shown in Fig. 4(b), together with τ_so_ and τ_ϕ_, which are deduced from τ_so_(τ_ϕ_) = *l*_so_(*l*_ϕ_)^2^/*D* with diffusion constant *D* given by *D* = v_F_^2^ τ/3. Here *v*_F_ is the Fermi velocity and τ is the momentum scattering time given by τ = μ*m* ^*^/*e* (*m* ^*^: effective electron mass) with *m* ^*^ = 0.023 *m*_e_ (*m*_e_: electron mass). We also note that *l*_ϕ_ > *W*, which is required for a one-dimensional weak antilocalization condition, is satisfied as shown in Fig. 4(b). As *V*_g_ increases, *l*_so_ decreases and *l*_ϕ_ increases, reaching a crossover at *V*_g_ ~ 0.5 V. This corresponds to the gate voltage at which a crossover from weak localization to weak antilocalization occurs. The decreasing *l*_so_ accompanied by a rapid decrease in τ_so_ demonstrates that the spin-orbit relaxation length is tuned significantly by the electric field induced by the gate voltage.

## Discussion

**Figure 5:**
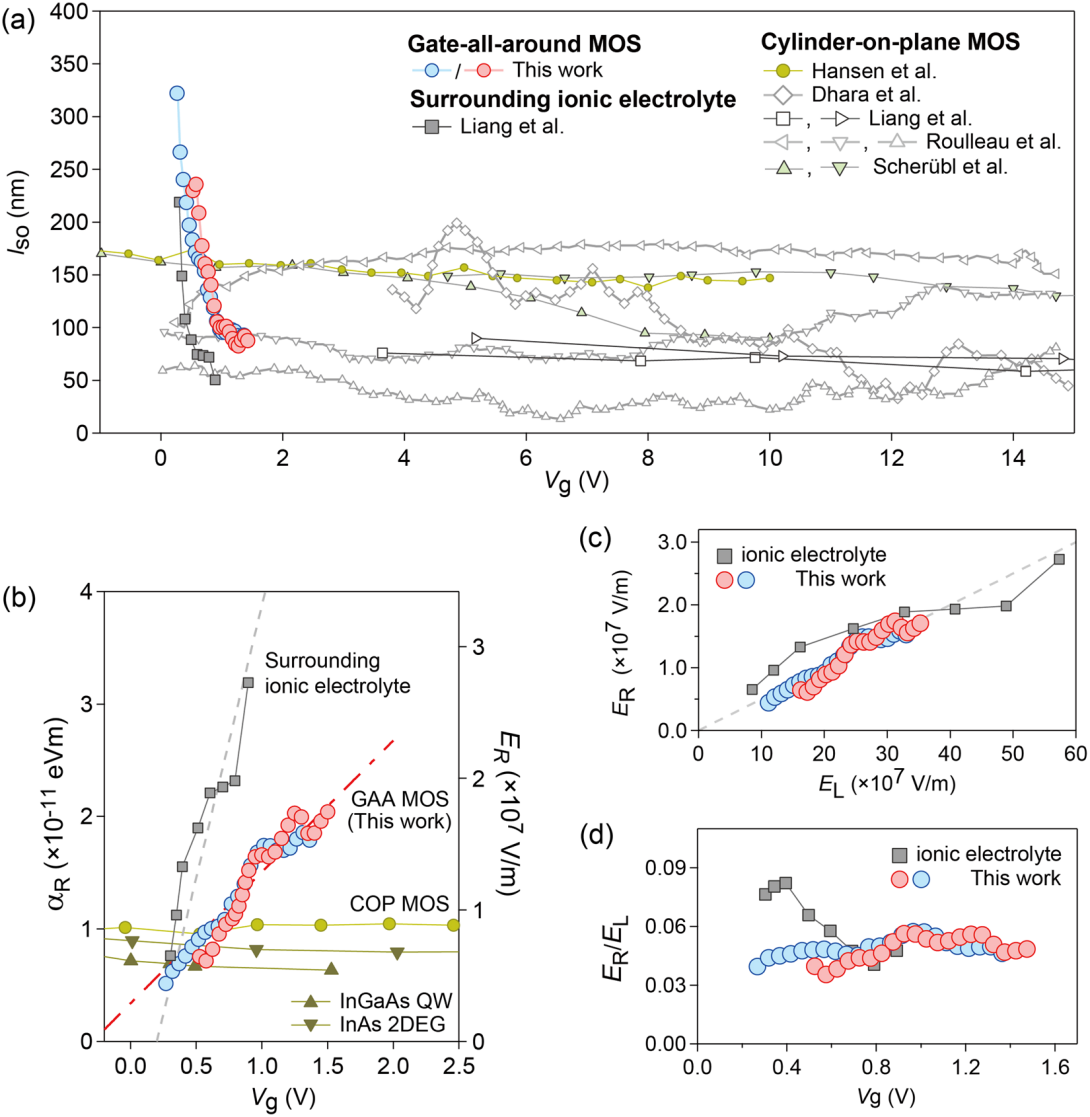
(**a**) Comparison of *V*_g_ dependence of *l*_so_ in our device and those in previously reported InAs nanowire devices. They are categorized as having GAA geometry and back- and/or top-gate (cylinder-on-plane) geometry. (**b**) Rashba parameter α_R_ and associated electric field *E*_R_ plotted as a function of *V*_g_ for our GAA InAs nanowire MOSFET, an InAs nanowire device using electrolyte^17^, an InAs nanowire device using a back-gate with cylinder-on-plane (COP) geometry^13^, InGaAs QW^8^, and InAs 2DEG used to develop a spin FET^7^. Data shown with red and blue symbols were obtained from measurement runs for the first cooling and for the cooling after 6 months interval in Fig. 3. (**c**) *E*_R_ as a function of *E*_L_ for our device and that in ref. 17. (**d**) *E*_R_ to *E*_L_ ratio as a function of *V*_g_ for our device and that in ref. 17.

We in turn compare the *V*_g_ tunability of *l*_so_ obtained for our device with those already reported for other InAs nanowire FETs^13–18^. As is clearly seen in Fig. 5(a), where *l*_so_ is plotted against *V*_g_, our GAA MOS-type device shows superior *V*_g_ tunability; *l*_so_ is modulated several times in a *V*_g_ range an order of magnitude smaller than that used to operate back or top-gated (cylinder-on-plane) InAs nanowires^13–16, 18^, indicating that our GAA MOSFET can offer much lower power consumption than conventional nanowire MOSFETs. The tunability for our device also reaches a high level comparable to the previously reported best controllability obtained for an InAs nanowire device operated with electrolyte gating^17^. It is noteworthy that such high *V*_g_ tunability is achieved for a MOSFET, which has an advantage of easier and faster operation than ion-gated devices particularly in temperature-variable measurements. This is because ion-gated devices typically require the temperature to be increased to change the carrier density for ion polarization^41^, which itself requires a long time to stabilize^23^. These types of devices sometimes take more than ten hours for temperature variation to minimize sample electrochemical degrading^42^.

Using experimentally extracted *l*_so_, we calculated the Rashba coupling parameter α_R_ and corresponding electric field *E*_R_. Here α_R_ is given by ${\alpha }_{R}=\frac{{\hslash }^{2}}{2{m}^{\ast }{l}_{so}}={\alpha }_{0}e{E}_{R}$, where *ħ* is the reduced Planck constant and α_0_ is the Rashba coefficient of bulk InAs α_0_ = 1.17 nm^2^ (ref. 43). Figure 5(b) shows α_R_ and *E*_R_ as a function of *V*_g_. The red and blue circles indicate data obtained for the first cooling [with a mobility of 1200 cm^2^/Vs, as shown in Fig. 3(a,c and d)] and for the cooling carried out with an interval of 6 months [with a mobility of 400 cm^2^/Vs, as shown in Fig. 3(a–d)]. Despite the long time interval and difference in mobility, the α_R_ and *E*_R_ values obtained from two measurements are in good agreement. When *V*_g_ is increased above the threshold voltage *V*_th_, α_R_ and *E*_R_ increase linearly as expected. A rapid increase in α_R_ up to *V*_g_ ~ 1.5 V provides Rashba parameter tunability reaching 1.2 × 10^−11^ eVm/V.

Figure 5(b) also summarizes the *V*_g_ tunability of the Rashba SOI extracted from various devices, where our device is compared with an ion-gated InAs nanowire device^17^, a back-gated cylinder-on-plane InAs nanowire^13^, and other two-dimensional FETs fabricated from strong SOI material^7, 8^. Here α_R_ is estimated by analysing the crossover from weak localization to weak anti-localization for the nanowire devices, and is extracted from beating patterns in magnetotransport for the two-dimensional FETs. While the *V*_g_ tunabilities of α_R_ and *E*_R_ for our sample are about a quarter of their counterparts for the ion-gated device^17^, they greatly exceed the values obtained for a conventional back-gated cylinder-on-plane InAs nanowire MOSFET^13^ as well as those obtained for two-dimensional FETs fabricated from III-V material^7, 8^.

We further investigate the ratio of the calculated electric field *E*_L_ expected from GAA geometry and the *E*_R_ value that is directly associated with the Rashba SOI. In the cylinder capacitance model, the charge line density *Q*_L_ and associated electric field *E*_L_ are given by,

3 $${Q}_{L}=\frac{C({V}_{g}-{V}_{fb})}{{L}_{g}}$$

4 $${E}_{L}=\frac{{Q}_{L}}{\pi ({\varepsilon }_{0}{\varepsilon }_{InAs})W}$$

where *C* is the cylindrical gate capacitance (see Method), *V*_fb_ is the gate voltage that gives flat band condition, *W* is the nanowire diameter, and ε_0_ and ε_InAs_ are the vacuum and relative permittivities. The slope of the *V*_g_ dependence of *E*_L_ is extracted for our device from these equations. We use *C* = 2.29 × 10^−14^ F, *L*_g_ = 3.3 μm, *W* = 100 nm for our sample. As for *V*_fb._ we use gate voltage given by the intercept of *E*_R_ = 0 for the Rashba measurements. The dash-dotted line in Fig. 5(b) tracing our data has a slope that is twenty times smaller than that for calculated *E*_L_. We then consider the ion-gated device described in ref. 17, where the authors adapted the same cylinder capacitance model to their device. We calculate *E*_L_ using the corresponding values shown in Supplementary Information in ref. 17 (*C* = 1.44 × 10^−14^ F, *L*_g_ = 2 μm, *W* = 25 nm). Their data are also traced by the dashed line with a slope twenty times smaller than *E*_L_ calculated for their device.

The inconsistency between *E*_L_ and *E*_R_ is pointed out in ref. 17, and they attributed it to electric field decay due to screening by the gate-induced charge in the nanowire channel^44^, also noting that this decay would appear similarly in GAA MOS-type nanowires. We consider that the inconsistency we found with our device is partly associated with this charge screening, which is mainly due to surface-state pinning^15^. We also mention that the field gradient on *V*_g_ can be reduced by trap states or interface states possibly incorporated in a gate insulator, which would act as a reservoir for gate-induced carriers^45, 46^, even though our device is expected to have less interface state density due to the insertion of an Al_2_O_3_ layer before HfO_2_ growth^47^. When we assume the presence of interface states located between the InAs surface and the Al_2_O_3_ gate insulator, the interface state density required to explain the dash-dotted line would be very large, reaching ~3 × 10^14^ eV^−1^cm^−2^ based on a model similar to that described in ref. 45. This unreasonably large value of the interface-state density itself suggests that our device is significantly affected by the charge screening effect.

To highlight the efficiency of our device, we compare *E*_L_, *E*_R_ and *E*_R_/*E*_L_ between the two devices. As expected from the device geometry, *E*_L_ for *V*_g_–*V*_fb_ of 1 V is calculated to be 4.0 × 10^8^ V/m for an ion-gated device (with their assumption of a Debye length of 1 nm (ref. 22), which corresponds to the gate insulator thickness in GAA geometry) and 1.0 × 10^8^ V/m for our device. It should be noted that, while we compare devices with different nanowire diameters, *E*_L_ is determined solely by the gate insulator material and gate geometry, and is thus inherently nearly independent of nanowire width. Although *E*_L_ for our device is about one quarter of its electrolyte counterpart, it is significant that a MOSFET has such a high *E*_L_ value owing to its thin high-κ gate dielectrics.

When *E*_R_ is plotted as a function of *E*_L_, instead of *V*_g_, as shown in Fig. 5(c), data from our MOS device and those from the ion-gated device fall on almost the same line. This consistency between totally different devices highlights the fact that our GAA device is fabricated as well as an ion-gated device as regards the gate-control efficiency that affects the Rashba SOI. Although the *E*_R_ to *E*_L_ ratio decreases to about 5% for both devices, our MOS device does not require any thermal cycle for gate voltage change unlike ion-gated device, and therefore enables *in-situ* continuous tuning of α_R_. Furthermore, the *E*_R_ to *E*_L_ ratio in our device is nearly independent of *V*_g_ [see Fig. 5(d)], thus ensuring more stable SOI operation by sweeping gate voltage.

The above results demonstrate that our GAA geometry with high-κ gate dielectrics has the Rashba SOI tuning efficiency close to the best value ever achieved, at the same time as enabling the continuous *in-situ* tuning due to the faster response of MOS design. We believe that these advantages will make our device a prototype nanoscale MOSFET for use in realizing practical spin control application.

## Method

InAs nanowires are grown by vapour-liquid-solid method using gold nanoparticles as catalysts^48^. For the gate dielectrics, we combined two high-κ gate dielectrics of Al_2_O_3_ (2 nm) and HfO_2_ (4 nm) grown by ALD. The growth of Al_2_O_3_ before HfO_2_ can improve the interface between InAs and gate dielectrics, which may reduce the interface state density in ALD-grown gate dielectrics^47^. As shown in Fig. 1(b), more than 90% of the channel length of our device is coated with a gate electrode. When we considered the contributions of ungated regions and deduced the corrected mobility as in refs 33 and 34, we found that the corrected mobility differs less than 5%, which allows us to disregard the contributions of the ungated regions. The sample was measured with a standard DC transport method or ac lock-in techniques at room temperature down to 1.5 K using a cryostat.

To obtain the gate capacitance, we used a standard cylindrical model. When a gate insulator with a thickness of *h* coats a nanowire with a radius *r* and length *L*_g_, the gate capacitance *C* is given by $C=\frac{2\pi {\varepsilon }_{0}{\varepsilon }_{h}{L}_{g}}{\mathrm{ln}(1+\frac{h}{r})}$, where ε_h_ is relative permittivity of the gate insulator. Since our device employed a double layer of high-κ gate dielectrics, Al_2_O_3_ and HfO_2_, we use the total gate capacitance *C*_tot_ given by $\frac{1}{{C}_{tot}}=\frac{1}{{C}_{1}}+\frac{1}{{C}_{2}}$, where ${C}_{1}=\frac{2\pi {\varepsilon }_{0}{\varepsilon }_{h1}{L}_{g}}{\mathrm{ln}(1+\frac{{h}_{1}}{r})}$ and ${C}_{2}=\frac{2\pi {\varepsilon }_{0}{\varepsilon }_{h2}{L}_{g}}{\mathrm{ln}(1+\frac{{h}_{2}}{(r+{h}_{1})})}$ with *h*_1_ being the thickness of Al_2_O_3_ (2 nm) and *h*_2_ being the thickness of HfO_2_ (4 nm). The values used for our calculation are *C*_tot_ = 2.29 × 10^−14^ F, *L*_g_ = 3.3 μm, *r* = 50 nm (*W* = 100 nm), and ε_InAs_ = 12.5.
